# Dr. Ralph A. Parsons

**Published:** 1891-07

**Authors:** George T. Eames

**Affiliations:** Committee for the Faculty


### DR. RALPH A. PARSONS.

DR. RALPH A. PARSONS, Professor of Materia Medica of the Boston Dental College, died April 3, 1891.

Dr. Parsons was born in Stratton, Vt. He graduated in medicine at the University of New York in 1888, and settled in

West Roxbury, Massachusetts, where he acquired an extensive practice.

At a special meeting of the Faculty of the Boston Dental College the following resolutions were passed:

WHEREAS, in the dispensations of Divine Providence, a most highly esteemed and able instructor in this college, Dr. Ralph A. Parsons, has been removed by death, therefore, be it

*Resolved*, That in the death of Dr. Parsons the Boston Dental College has sustained the loss of an earnest and enthusiastic worker, one whose able, scientific, and practical instructions were shown in the remarkable progress of the students under his charge.

It is, therefore, voted that a copy of these resolutions be spread upon the college records, in the leading medical and dental journals, and that a copy be also sent to his immediate relatives.

GEORGE T. EAMES, M.D., D.D.S.,

*Committee for the Faculty.*

